# Supplementary material for: Candidate targets of copy number deletion events across 17 cancer types
Source: Front Genet. 2023 Jan 16;13:1017657. doi: 10.3389/fgene.2022.1017657 (PMC9885371; doi:10.3389/fgene.2022.1017657)
Supplement: Supplementary file 1 [file DataSheet2.pdf]

Appendix 1. Significance across cancer types

Table S1: Significant genes in analyses (Data Sheet 1)

Table S2: The number of significant genes belonging to the three cancer driving gene sets and the one-sided Fisher exact test p value for each analysis (Data Sheet 1)

Appendix 2. Pathway enrichment and clustering

Table S3: Top 10 pathways enriched by the significant genes of 25 analyses (Data Sheet 1)

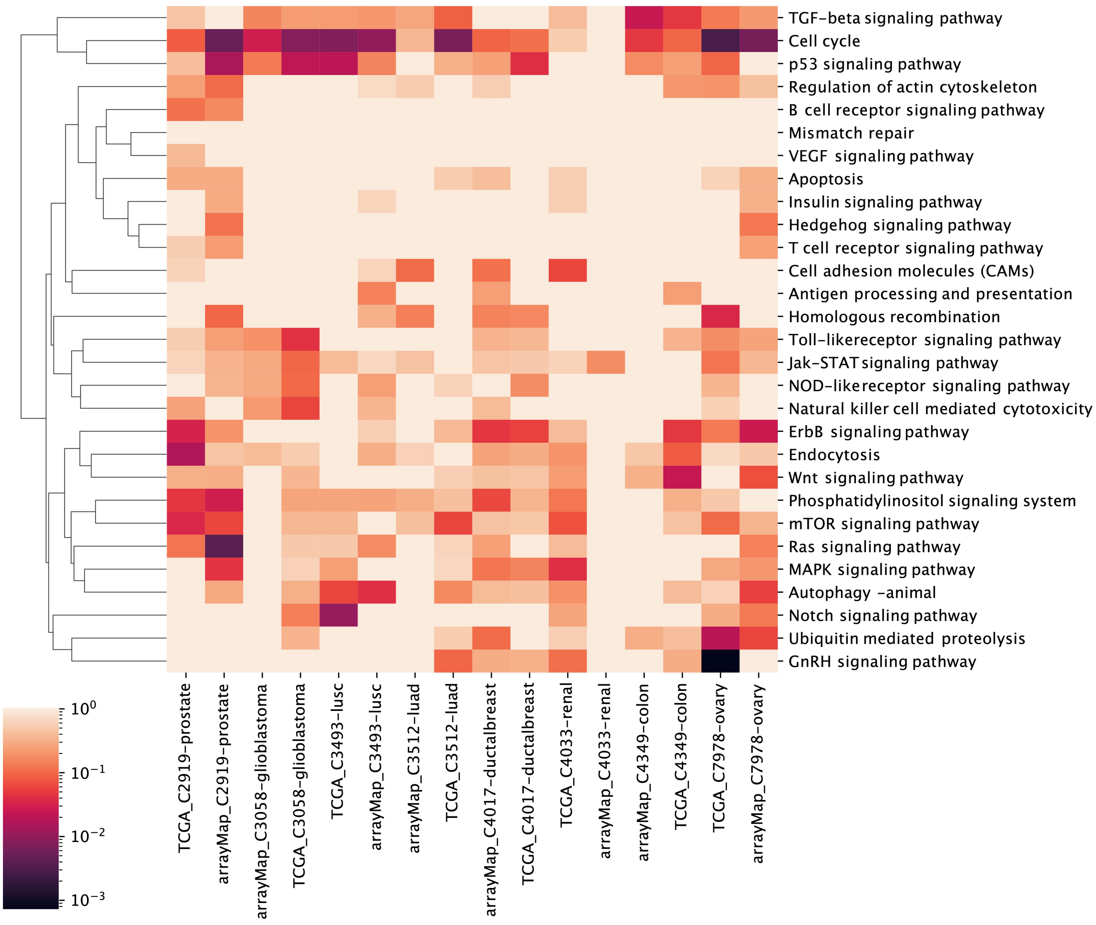

Figure S1: Clustering of genome-wide significance value of paired 8 cancer types with 29 cancer hallmark pathways

Appendix 3. Technical information of analyses and benchmarking sets

We have used GRCh38 (Ensembl Release 77) for the reference genome track and the gene CDS positions in the study.
Three independent driver sets include Bailey set [1] consisting of 299 genes from tumor exome analysis with experimental validation, the Dietlein set [2], including 461 genes from nucleotide context as well as the CGC set [3], including 724 genes from expert curation across multiple

cancer types. The three gene sets share 144 consensus genes and the number of genes private to one set ranges from 73 to 481 (Figure S5).

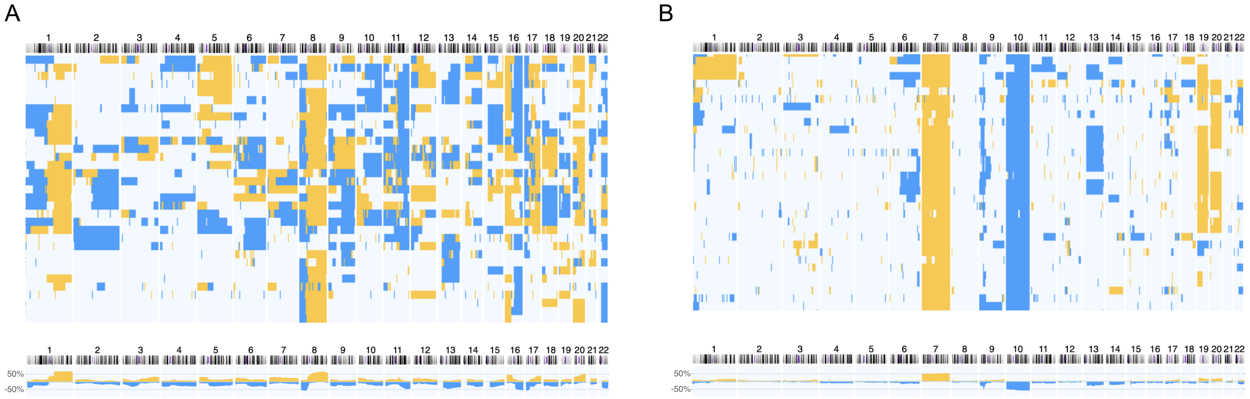

Figure S2: Genome-wide copy number landscape across multiple samples. A) Ductal Breast Carci- noma, NCIT:C4017; B) Glioblastoma, NCIT:C3058. In each cancer type, the top panel stacks 30 randomly selected individual samples' CNA profile and the lower panel indicates the aggregated CNA landscape by percentage of samples exhibiting gain (yellow) and loss (blue) across the genome.

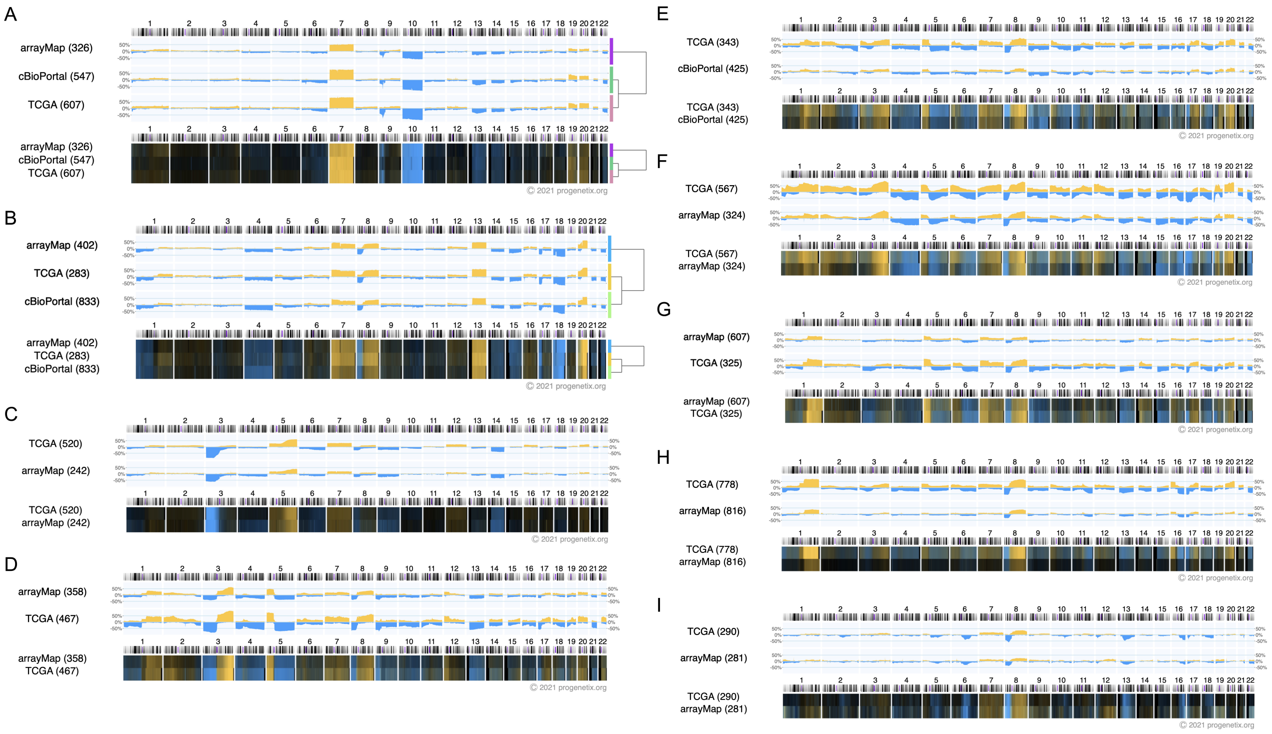

Figure S3: Genome-wide copy number landscape of nine cross-comparison cancer types A) glioblastoma (C3058); B) colon adenocarcinoma (C4349); C) clear cell renal cell carcinoma (C4033); D) lung squamous carcinoma (C3493); E) bladder urothelial carcinoma (C39851); F) ovary serous cystadenocarcinoma (C7978); G) lung adenocarcinoma (C3512); H) ductal breast carcinoma (C4017); I) prostate adenocarcinoma (C2919).

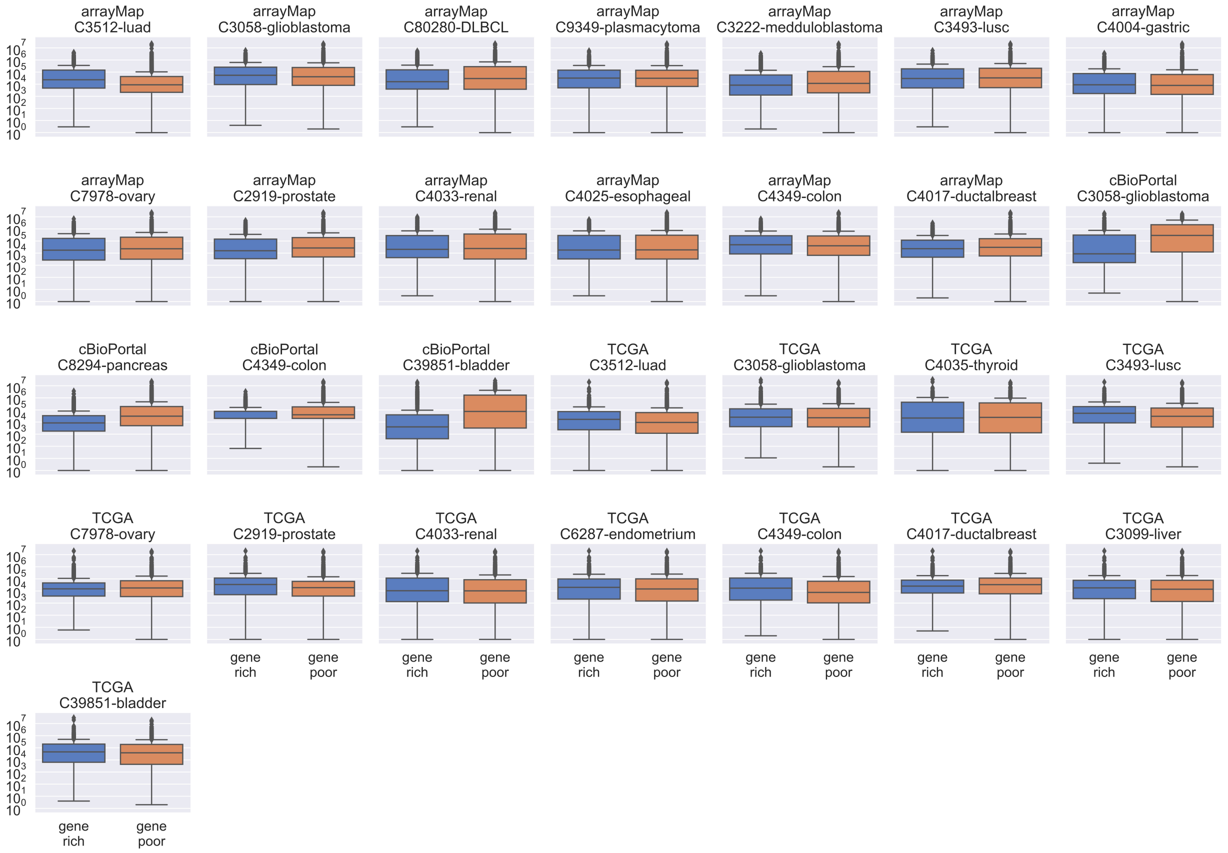

Figure S4: Number of segmental breakpoints in gene-rich and poor regions by analysis Most analyses have a nearly equal breakpoint density regardless of gene density but the four WES-derived (cBioPortal) analyses show clear segment sparsity in gene-poor regions.

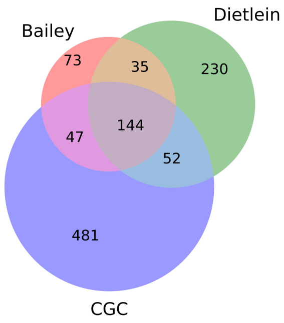

Figure S5: Overlap among the three cancer driving gene sets: Bailey, Dietlein and CGC [1,2,3]

References

1. M. H. Bailey, C. Tokheim, E. Porta-Pardo, S. Sengupta, D. Bertrand, A. Weerasinghe, A. Colaprico, M. C. Wendl, J. Kim, B. Reardon, et al., Comprehensive characterization of cancer driver genes and mutations, Cell 173 (2) (2018) 371–385.

2. F. Dietlein, D. Weghorn, A. Taylor-Weiner, A. Richters, B. Reardon, D. Liu, E. S. Lander, E. M. Van Allen, S. R. Sunyaev, Identification of cancer driver genes based on nucleotide context, Nature genetics 52 (2) (2020) 208–218.

3. Z. Sondka, S. Bamford, C. G. Cole, S. A. Ward, I. Dunham, S. A. Forbes, The cosmic cancer gene census: describing genetic dysfunction across all human cancers, Nature Reviews Cancer 18 (11) (2018) 696–705.
